# Risk of selected immune-mediated inflammatory diseases after solid organ transplantation in a Korean nationwide cohort

**DOI:** 10.1016/j.jdin.2026.05.020

**Published:** 2026-06-01

**Authors:** Jae Joon Jeon, You Hyun Kim, Hyunsoo Son, Yeon-Woo Heo, Jae Hyeon Seo, Min Chul Ha, Seung-Won Jung, Ju Yeong Lee, Hunju Lee, Sang Baek Koh, Hyebin Song, Jee Hyun Kong, Eunbee Choi, Hajeong Kim, Shinwon Hwang, Chul S. Hyun, Daniel S. Hippe, Song Youn Park, Jae Il Shin, Solam Lee, Eung Ho Choi

**Affiliations:** a Department of Dermatology, Yonsei University Wonju College of Medicine, Wonju, Republic of Korea; b Institute of Evidence Based Medicine, Yonsei University Wonju College of Medicine, Wonju, Republic of Korea; c Department of Bioengineering, College of Engineering, University of Washington, Seattle, Washington; d Washington National Biomedical Research Center, Seattle, Washington; e Fred Hutchinson Cancer Center, Seattle, Washington; f Department of Preventive Medicine, Yonsei University Wonju College of Medicine, Wonju, Republic of Korea; g Division of Hematology-Oncology, Department of Internal Medicine, Yonsei University Wonju College of Medicine, Wonju, Republic of Korea; h Department of Dermatology, Cutaneous Biology Research Institute, Gangnam Severance Hospital, Yonsei University College of Medicine, Seoul, Republic of Korea; i Department of Dermatology, Cutaneous Biology Research Institute, Severance Hospital, Yonsei University College of Medicine, Seoul, Republic of Korea; j Section of Digestive Diseases, Yale School of Medicine, New Haven, Connecticut; k Department of Dermatology, University of Washington, Seattle, Washington; l Department of Pediatrics, Yonsei University College of Medicine, Seoul, Republic of Korea; m Severance Underwood Meta-Research Center, Institute of Convergence Science, Yonsei University, Seoul, Republic of Korea; n Galleria Dermatology Clinic, Seoul, Republic of Korea

**Keywords:** alopecia areata, epidemiology, immune-mediated inflammatory disease, immunology, incidence, lupus, psoriasis, risk, solid organ transplant, transplantation, vitiligo

*To the Editor:* Solid organ transplant recipients (SOTRs) require lifelong, high-dose immunosuppressive therapy, resulting in an increased risk of immune-related comorbidities. While a prior study reported an increase in specific immune-mediated inflammatory diseases (IMIDs) among pediatric SOTRs, evidence characterizing these risks in the general SOTR population remains limited.^1^ Our study aimed to investigate the incidence of selected IMIDs in SOTRs utilizing the Korea Healthcare Bigdata Platform.

This nationwide cohort study was performed using integrated data from multiple Korean government agencies from January 1, 2012, to December 31, 2021. The SOTR cohort (*n* = 14,577) was propensity score-matched to a control cohort at a 1:1 ratio based on demographics and comorbidities (Table I, Supplementary Fig 1, available via Mendeley at https://data.mendeley.com/datasets/tp59mymyc7/1). Patients who underwent more than 1 transplantation were excluded. The main outcome was the risk of IMIDs in the SOTR cohort compared with control cohort. Multivariable Cox proportional hazards models were used to estimate adjusted hazard ratios for 15 predefined IMID outcomes. In sensitivity analyses, Poisson regression was used to estimate incidence rate ratios for each IMID between cohorts.^2^ More details provided in Supplementary Methods and Tables, available via Mendeley at https://data.mendeley.com/datasets/tp59mymyc7/1.

**Table I:** Demographic characteristics of the solid organ transplant recipient cohort and the control cohort before and after propensity score-matching

Characteristics	Prematching, patients, no. (%)			Postmatching, patients, no. (%)		
	SOTR (*N* = 18,065)	Control (*N* = 796,276)	SMD	SOTR (*N* = 14,577)	Control (*N* = 14,577)	SMD
Age (y), mean (SD)	58.30 (11.18)	56.32 (16.40)	0.141	60.68 (9.90)	61.44 (11.03)	0.054
Sex, *n* (%)			0.370			0.099
Male	11,897 (65.86)	382,361 (48.02)		9455 (64.86)	10,157 (69.68)	
Female	6168 (34.14)	413,915 (51.98)		5122 (35.14)	4420 (30.32)	
Insurance type, *n* (%)			0.354			0.051
Standard	15,505 (85.83)	763,381 (95.87)		12,906 (88.54)	12,695 (87.09)	
Medicaid	2560 (14.17)	32,895 (4.13)		1671 (11.46)	1882 (12.91)	
Area of residence, *n* (%)			0.009			0.005
Urban area	7998 (44.27)	348,674 (43.79)		6389 (43.83)	6424 (44.07)	
Rural area	10,067 (55.73)	447,602 (56.21)		8188 (56.17)	8153 (55.93)	
Underlying disease, *n* (%)						
Congestive heart failure	2711 (15.01)	29,478 (3.70)	0.396	1862 (12.77)	2275 (15.61)	0.099
Liver disease	5373 (29.74)	9388 (1.18)	0.861	4647 (31.88)	5457 (37.44)	0.167
Chronic kidney disease	11,746 (65.02)	15,254 (1.92)	1.799	8421 (57.77)	7611 (52.21)	0.158

*SMD*, Standardized mean difference; *SOTR*, solid organ transplant recipient.

The SOTR cohort comprised 7887 kidney, 5458 liver, 648 heart, and 584 other organ transplant recipients. No increased risk of most IMIDs was observed among SOTRs (Fig 1, Supplementary Fig 2, Table III, available via Mendeley at https://data.mendeley.com/datasets/tp59mymyc7/1). However, SOTRs exhibited a lower risk of alopecia areata (AA) than controls (AA; adjusted hazard ratio, 0.55; 99% CI, 0.32-0.97). In the sensitivity analysis using Poisson regression (Supplementary Fig 3, available via Mendeley at https://data.mendeley.com/datasets/tp59mymyc7/1), risks were lower for both AA (incidence rate ratio, 0.57; 95% CI, 0.43-0.77) and psoriasis vulgaris (psoriasis vulgaris; incidence rate ratio, 0.59; 95% CI, 0.42-0.82). Subgroup analyses stratified by demographics, organ type, donor type, and ABO incompatibility revealed no significant association with the risk of IMIDs (Supplementary Figs 4 to 7, available via Mendeley at https://data.mendeley.com/datasets/tp59mymyc7/1). Although statistical power for most IMIDs was <0.8, it exceeded 0.8 for AA in the Poisson regression model (Supplementary Figs 8 and 9, available via Mendeley at https://data.mendeley.com/datasets/tp59mymyc7/1).

**Fig 1:**
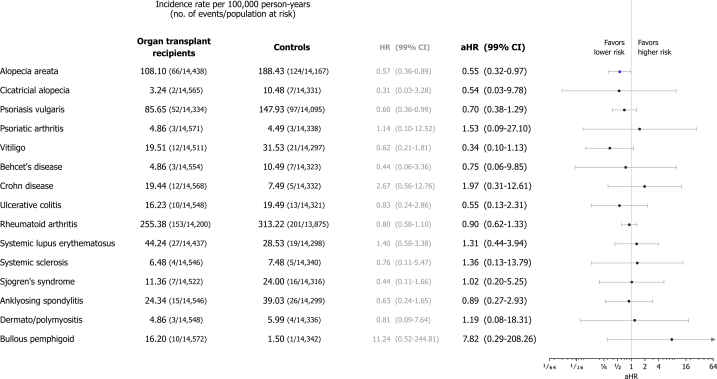
Risks of incident immune-mediated inflammatory diseases in the solid organ transplant recipient cohort compared with the control cohort. The confidence interval was set at 99.67% but is presented as 99% for simplicity.

Over a mean 4.2-year follow-up, most IMID risks in SOTRs were not increased compared to controls. Our results are consistent with the United States study reporting a decreased incidence of AA.^3^ Incorporating the sensitivity analyses, our findings suggest that SOTRs may have a reduced risk of AA and psoriasis vulgaris. Given that T cells play a critical role in both the pathogenesis of IMIDs and transplant immunology, high-dose immunosuppressive therapy in SOTRs may contribute to the observed lower risk of these IMIDs.^4^ However, because the risk of bullous pemphigoid did not decrease despite the frequent use of mycophenolate mofetil in SOTRs, these findings cannot be explained solely by pharmacological factors. Moreover, variations in healthcare utilization among SOTRs may influence the diagnosis of IMIDs. Taken together with the increasing life expectancy of SOTRs, future studies are warranted to focus on the development of IMIDs.^5^ Limitations include a homogeneous ethnicity, lack of information regarding immunosuppressive regimens, potential detection bias under claim-based study design, and a limited observation duration. Lastly, the limited number of events and subsequent lower statistical power preclude definitive conclusions. Nevertheless, our population-based study offers comprehensive clinical insights into the risk of IMIDs among SOTRs.

## Conflicts of interest

None disclosed.

## References

[1] Marcus N., Amir A.Z., Grunebaum E. (2018). De novo allergy and immune-mediated disorders following solid-organ transplantation-prevalence, natural history, and risk factors. J Pediatr.

[2] Zhang B., Wu Q., Jhaveri R. (2026). Long COVID associated with SARS-CoV-2 reinfection among children and adolescents in the omicron era (RECOVER-EHR): a retrospective cohort study. Lancet Infect Dis.

[3] Roman I., Aristizabal M.A., Leeaphorn N., Tolaymat L.M., Pincelli T.P. (2025). Alopecia areata in solid organ transplant recipients: a retrospective analysis. JAAD Int.

[4] Sun L., Su Y., Jiao A., Wang X., Zhang B. (2023). T cells in health and disease. Signal Transduct Target Ther.

[5] Graham C.N., Watson C., Barlev A., Stevenson M., Dharnidharka V.R. (2022). Mean lifetime survival estimates following solid organ transplantation in the US and UK. J Med Econ.

